# Supplementary material for: Association of plasma miRNAs with early life performance and aging in dairy cattle
Source: PLoS One. 2023 Jul 10;18(7):e0288343. doi: 10.1371/journal.pone.0288343 (PMC10332601; doi:10.1371/journal.pone.0288343)
Supplement: S1 File — (PDF) [file pone.0288343.s002.pdf]

S2 Table 1: Animal performance traits, distribution and independent fixed effects analyzed with GLMMs.

Dependent variable	Distribution	Independent fixed effects[1]
AFS	Normal	Month born<br>Cohort<br>ADG to 1 yr. (kg/day)
S/C	Poisson	Cohort
No. infections	Poisson	Cohort
Yr. 1 weight	Normal	Age (days) at measurement<br>Birth weight (kg)<br>ADG to weaning (kg/day)
ADG-wean	Normal	Weaning age (days)<br>Month born<br>Cohort<br>Birth weight (kg)<br>Dam age (days)<br>No. prior illnesses

[1]In addition to the fixed effects listed, all models included miRNA level and age at sampling (as fixed effects) and animal ID with associated pedigree as a random effect to account for additive genetic variation. Month born and cohort are factors while other fixed effects are continuous.

S2 Table 2: miRNAs that were significantly different between Group C and Group G in a t-test (P<0.05), based on array data, with mean and SE of each group.

miRNA[1]	P-value	Mean Group G (SE)	Mean Group C (SE)
**bta-miR-126-3p**	0.019	0.872 (0.173)	2.012 (0.148)
hsa-let-7a-5p	0.046	0.336 (0.076)	0.687 (0.134)
hsa-let-7c-5p	0.024	0.356 (0.028)	0.501 (0.041)
hsa-miR-24-3p	0.037	9.445 (0.899)	6.63 (0.554)
**hsa-miR-425-3p**	0.023	0.25 (0.025)	0.169 (0.012)
hsa-miR-133a-3p	0.011	0.126 (0.045)	0.068 (0.016)

[1]miRNAs that were chosen for qPCR validation are in bold.

S2 Table 3: miRNAs that were significantly associated with a trait of interest (GLMM: P<0.05), based on array data, with the predicted effects from the GLMMs.

Test	miRNA[1]	P-value	Model Predicted Effect
ADG-wean	hsa-let-7c-5p	0.029	0.08
Age 1st service	bta-let-7e	0.014	-22.46
	bta-miR-106a	0.010	-29.60
	bta-miR-152	0.040	17.58
	bta-miR-16b	0.020	-24.69
	bta-miR-195	0.004	23.33
	bta-miR-199a-5p	0.011	33.06
	bta-miR-199c	0.002	28.06
	bta-miR-200a	0.002	25.93
	bta-miR-2284ab	0.007	-35.28

Test	miRNA[1]	P-value	Model Predicted Effect
Age 1st service	bta-miR-27a-3p	0.005	23.29
	bta-miR-2904	0.049	-22.92
	bta-miR-423-3p	0.007	-24.97
	bta-miR-425-5p	0.027	-31.36
	bta-miR-455-3p	0.007	22.24
	bta-miR-93	0.017	-34.92
	bta-miR-99a-5p	0.044	19.95
	cfa-miR-411	0.034	-30.40
	gga-miR-17-5p	0.009	-32.22
	gga-miR-193b-3p	0.006	26.04
	ggo-miR-424	0.003	29.49
	hsa-let-7a-5p	0.009	-23.64
	hsa-let-7c-5p	0.008	-25.19
	hsa-let-7g-5p	0.011	-29.39
	hsa-miR-103a-3p	0.013	-25.43
	hsa-miR-125b-5p	0.002	26.65
	**hsa-miR-127-3p**	0.027	22.20
	hsa-miR-130a-3p	0.002	29.37
	hsa-miR-132-3p	<0.001	28.70
	hsa-miR-139-5p	0.006	-45.59
	hsa-miR-145-5p	0.004	22.48
	hsa-miR-148a-3p	0.002	26.29
	hsa-miR-15b-5p	0.023	-20.82
	hsa-miR-185-5p	0.026	-25.97
	hsa-miR-186-5p	0.046	16.35
	hsa-miR-191-5p	0.023	-32.00
	hsa-miR-199a-3p	0.006	27.69
	hsa-miR-200c-3p	0.008	21.96
	hsa-miR-205-5p	0.004	22.01
	hsa-miR-20a-5p	0.016	-28.99
	hsa-miR-25-3p	0.035	-27.25
	**hsa-miR-27b-3p**	0.006	23.58
	hsa-miR-369-3p	0.009	20.33
	hsa-miR-877-5p	0.007	26.94
	lca-miR-16	0.043	-23.43
	mmu-miR-497a-5p	0.005	33.74
	pma-miR-23b	0.020	17.76
	ssc-miR-15a	0.030	-23.75
No. infections	**bta-miR-126-3p**	0.016	0.45
	bta-miR-1260b	0.023	0.24
	bta-miR-1343-3p	0.044	0.29

Test	miRNA[1]	P-value	Model Predicted Effect
No. infections	bta-miR-21-5p	0.038	0.22
	bta-miR-2478	0.005	0.36
	bta-miR-2904	0.030	0.26
	**bta-miR-363**	0.046	0.23
	bta-miR-375	0.015	-0.63
	hsa-let-7d-5p	0.037	0.32
	hsa-let-7f-5p	0.018	0.32
	hsa-miR-139-5p	0.027	0.23
	hsa-miR-29c-3p	0.029	-0.81
	**hsa-miR-34a-5p**	0.001	0.45
	hsa-miR-376b-3p	0.013	-0.66
	hsa-miR-377-3p	0.045	-0.45
	hsa-miR-4286	0.003	0.34
	hsa-miR-532-5p	0.005	0.36
	mdo-miR-215	0.017	-0.41
	ssc-miR-181a	0.004	0.30
Yr. 1 weight	bta-miR-181b	0.041	22.19
	bta-miR-29d-3p	0.030	-21.43
	bta-miR-301b	0.005	-29.63
	bta-miR-339b	0.027	-21.80
	**bta-miR-363**	0.029	51.11
	hsa-let-7b-5p	0.023	23.19
	hsa-miR-128-3p	0.027	-27.81
	hsa-miR-223-3p	0.015	-23.80
	**hsa-miR-27b-3p**	0.043	-21.49
	hsa-miR-29b-3p	0.022	-22.17
	hsa-miR-30b-5p	0.042	-21.30
	**hsa-miR-30c-5p**	0.024	-25.47
	hsa-miR-326	0.022	-23.04
	**hsa-miR-34a-5p**	0.010	42.24
	hsa-miR-362-5p	0.023	24.13
	hsa-miR-379-5p	0.018	25.47
	**hsa-miR-425-3p**	0.020	-33.06
	hsa-miR-505-3p	0.018	45.73
	**oar-miR-154b-5p**	0.010	25.66
	ssc-miR-1468	0.007	43.44
	ssc-miR-224	0.022	22.05
	**xla-miR-142**	0.008	-25.98

[1]miRNAs that were chosen for qPCR validation are in bold.
